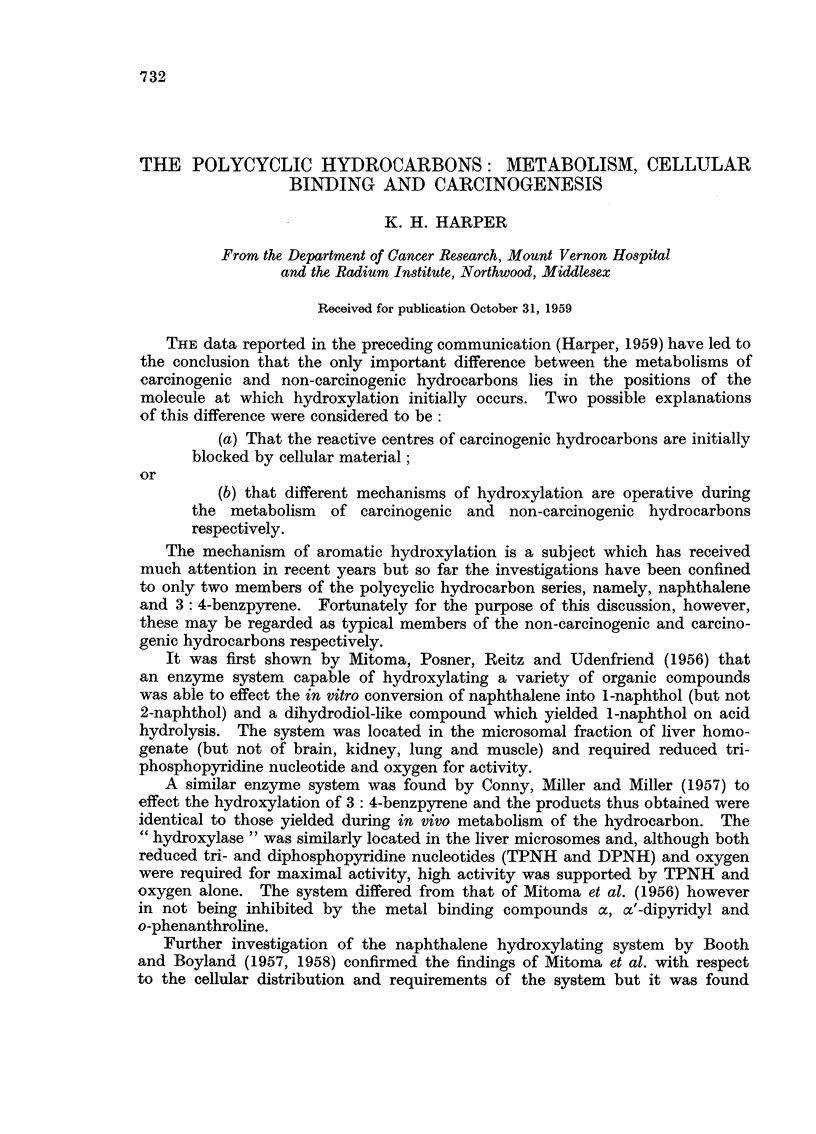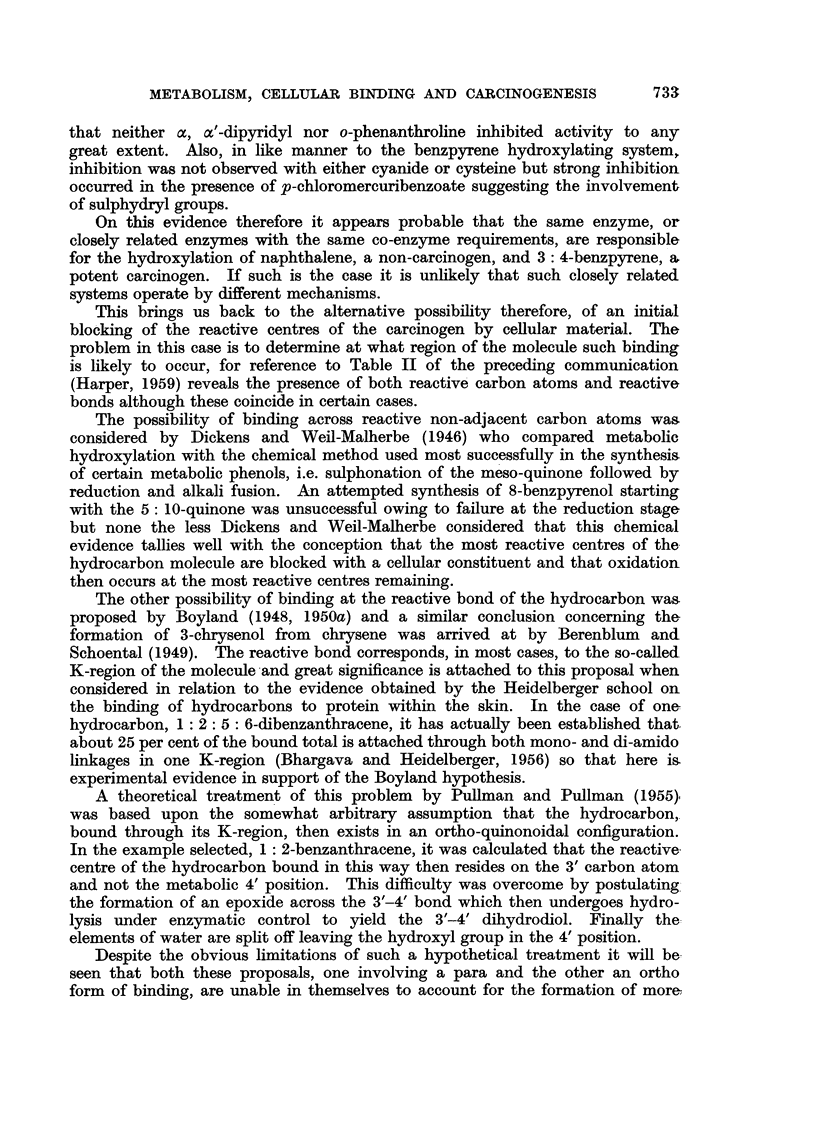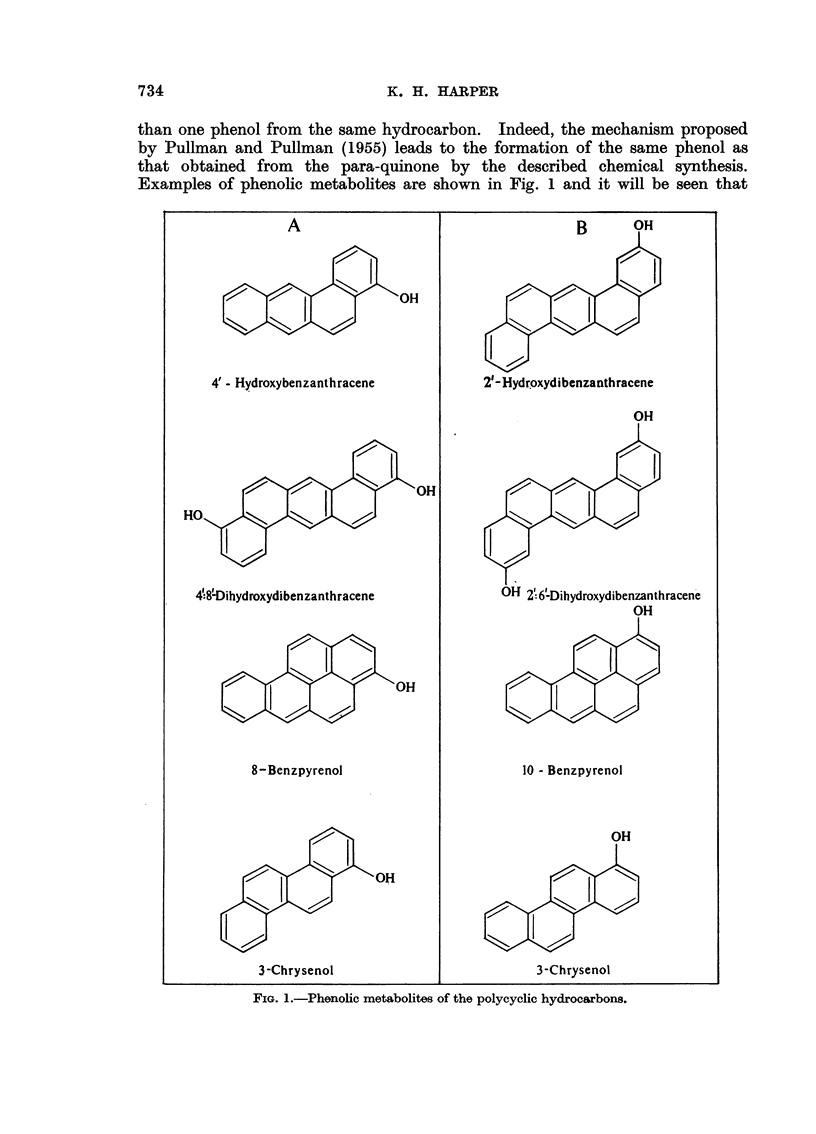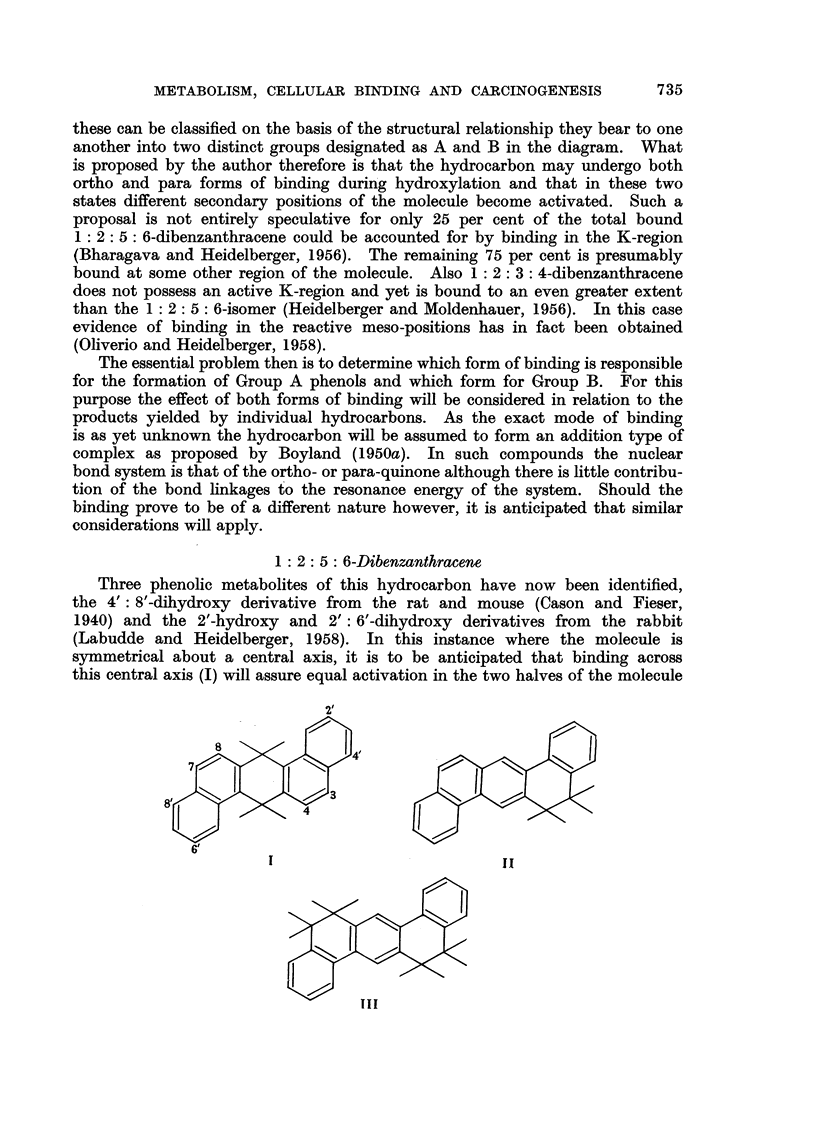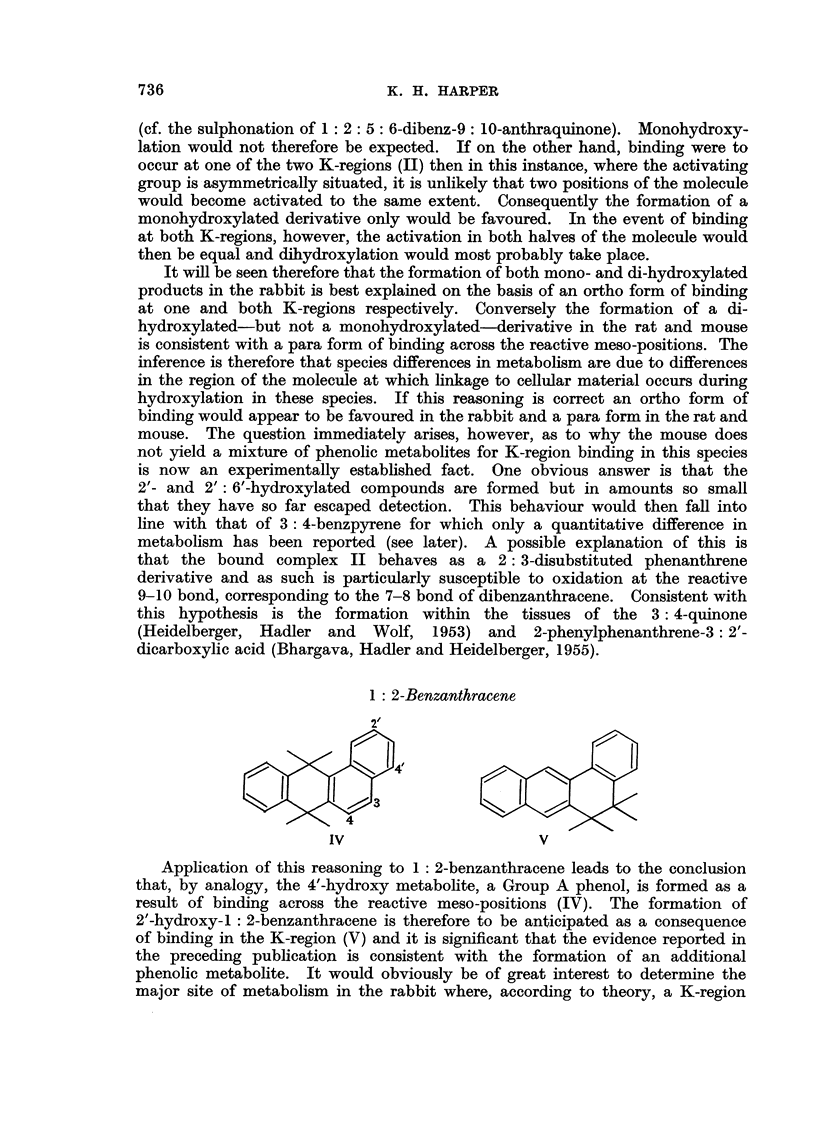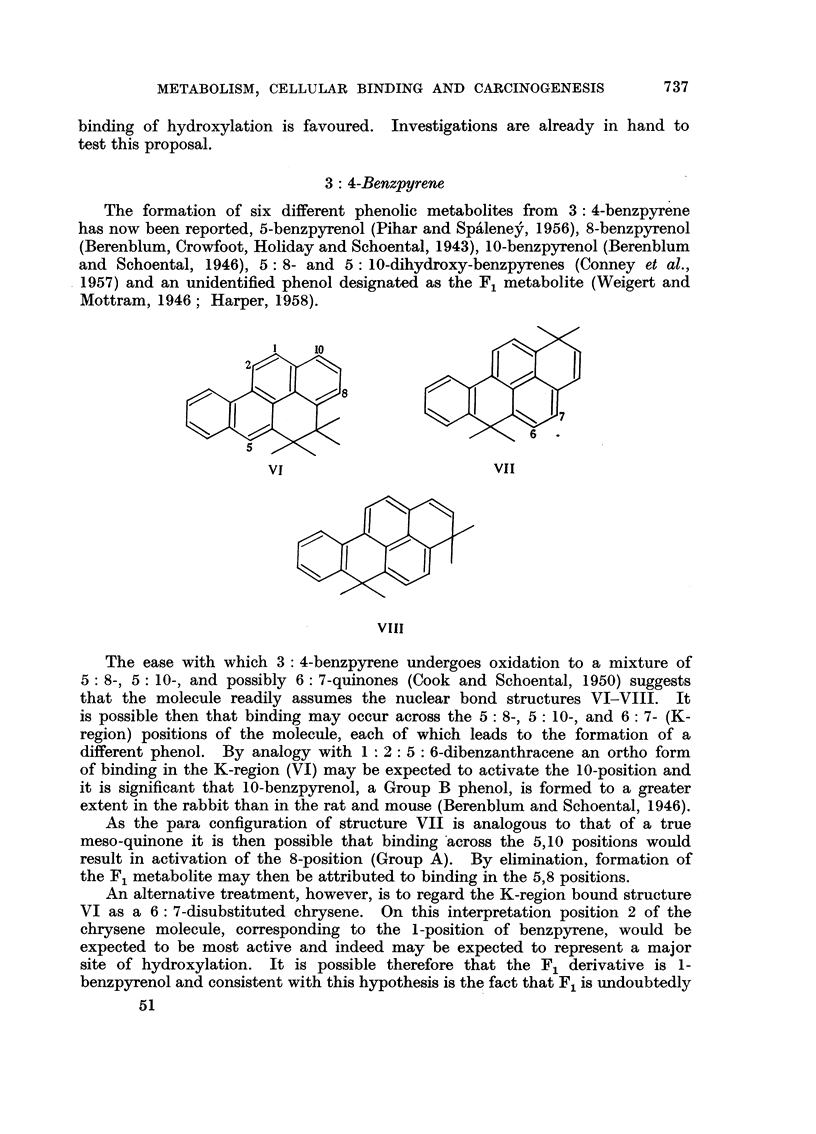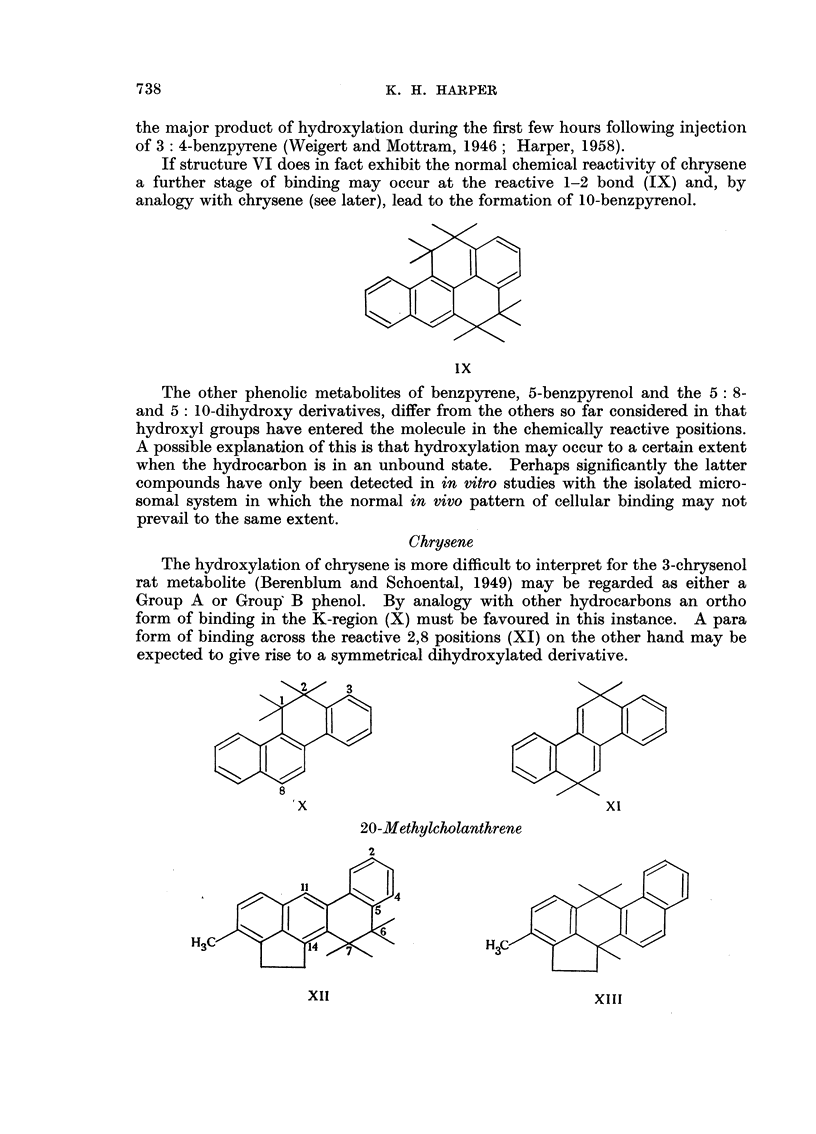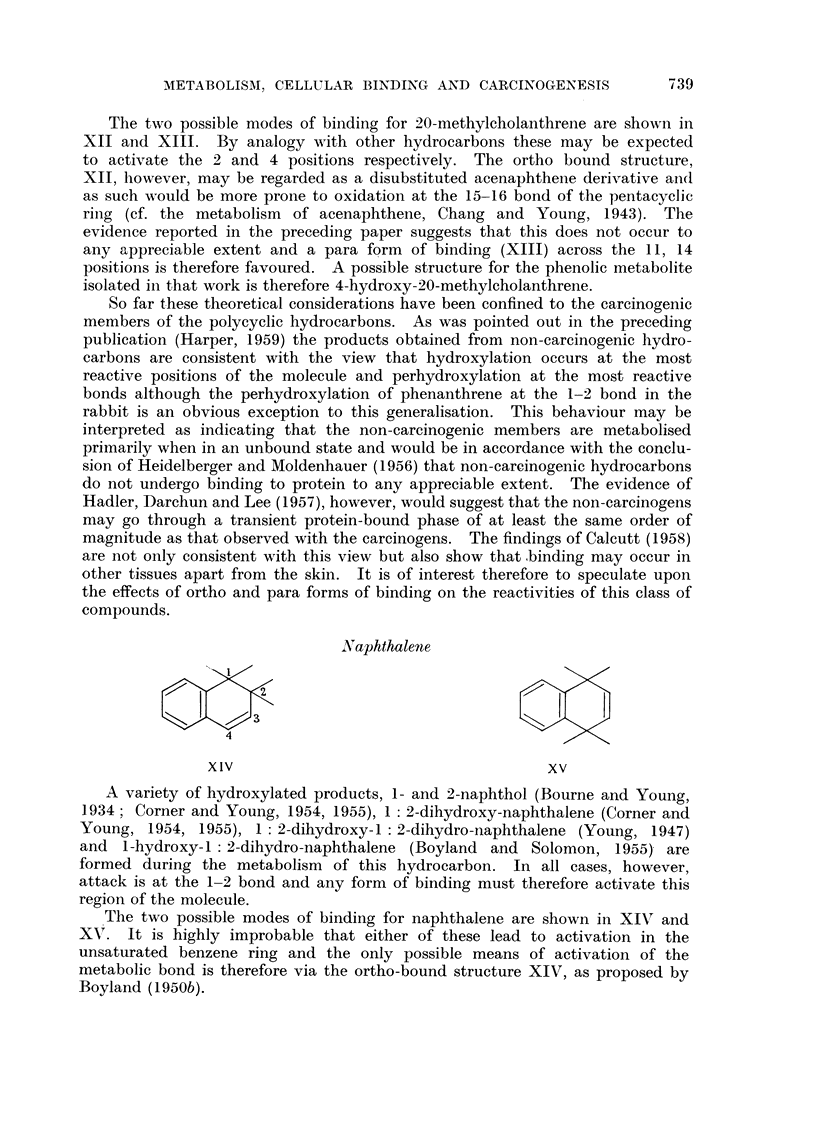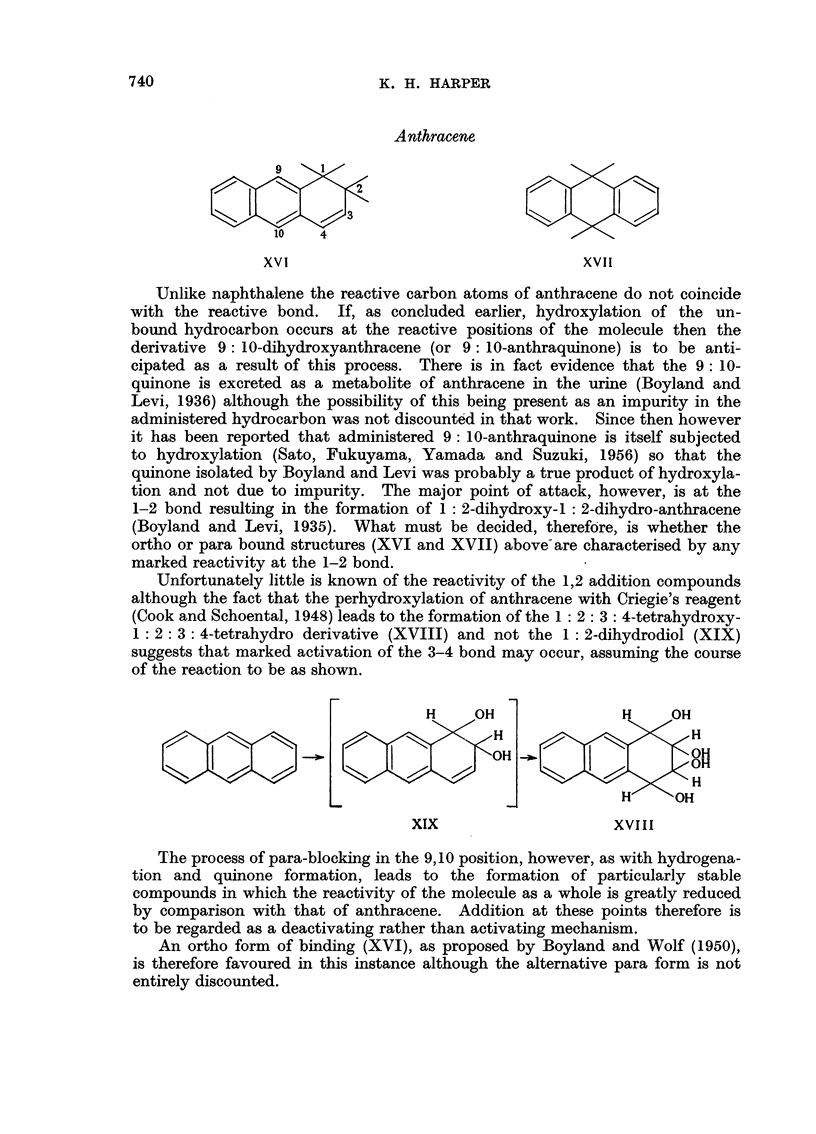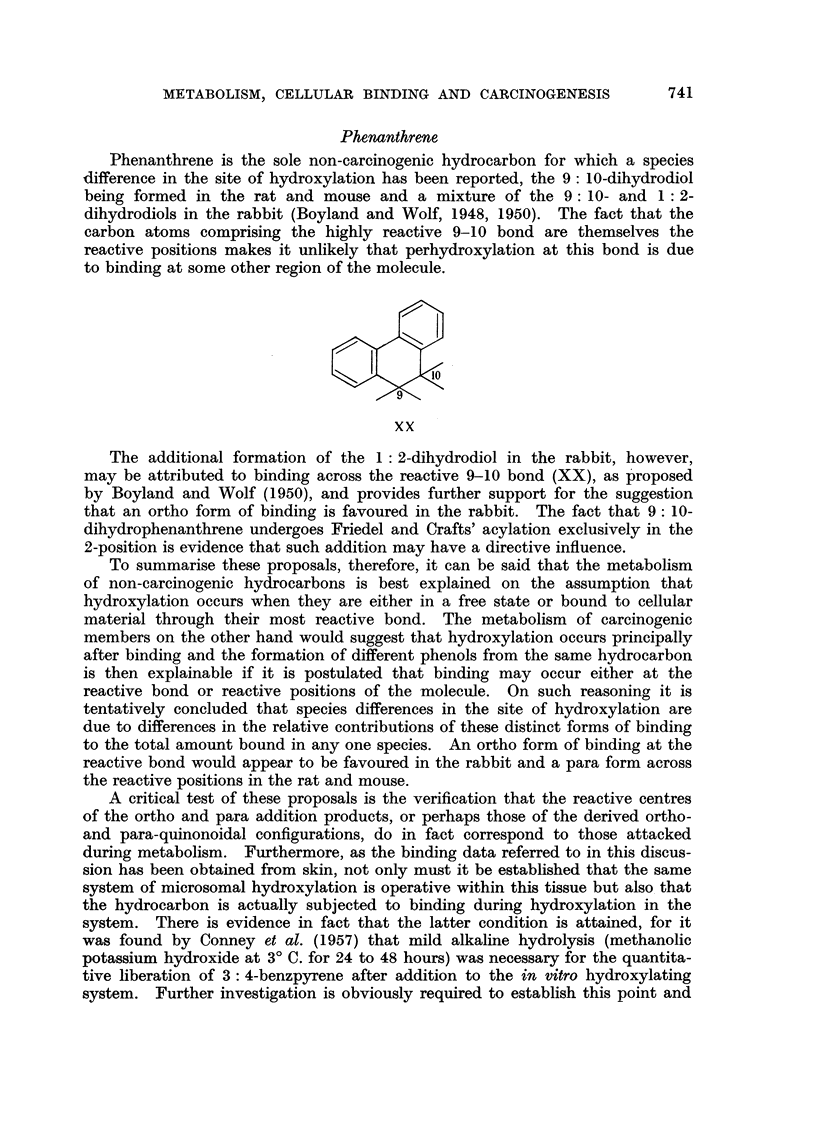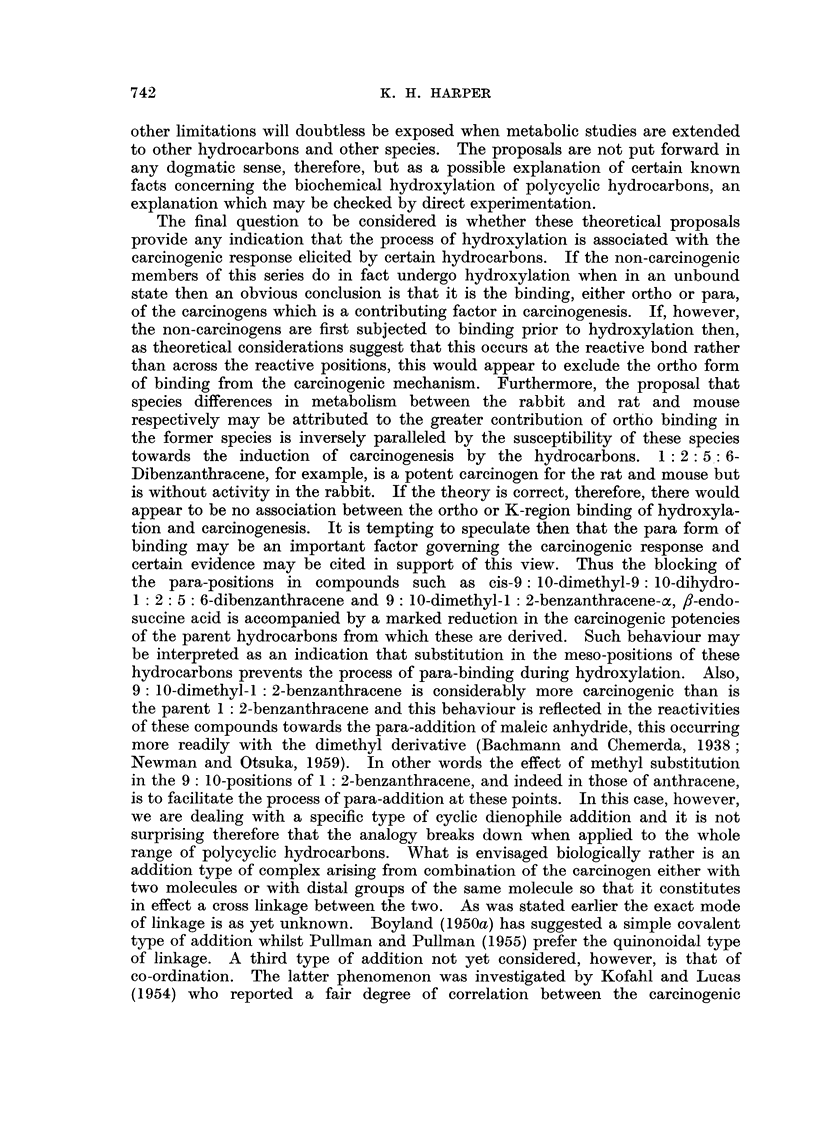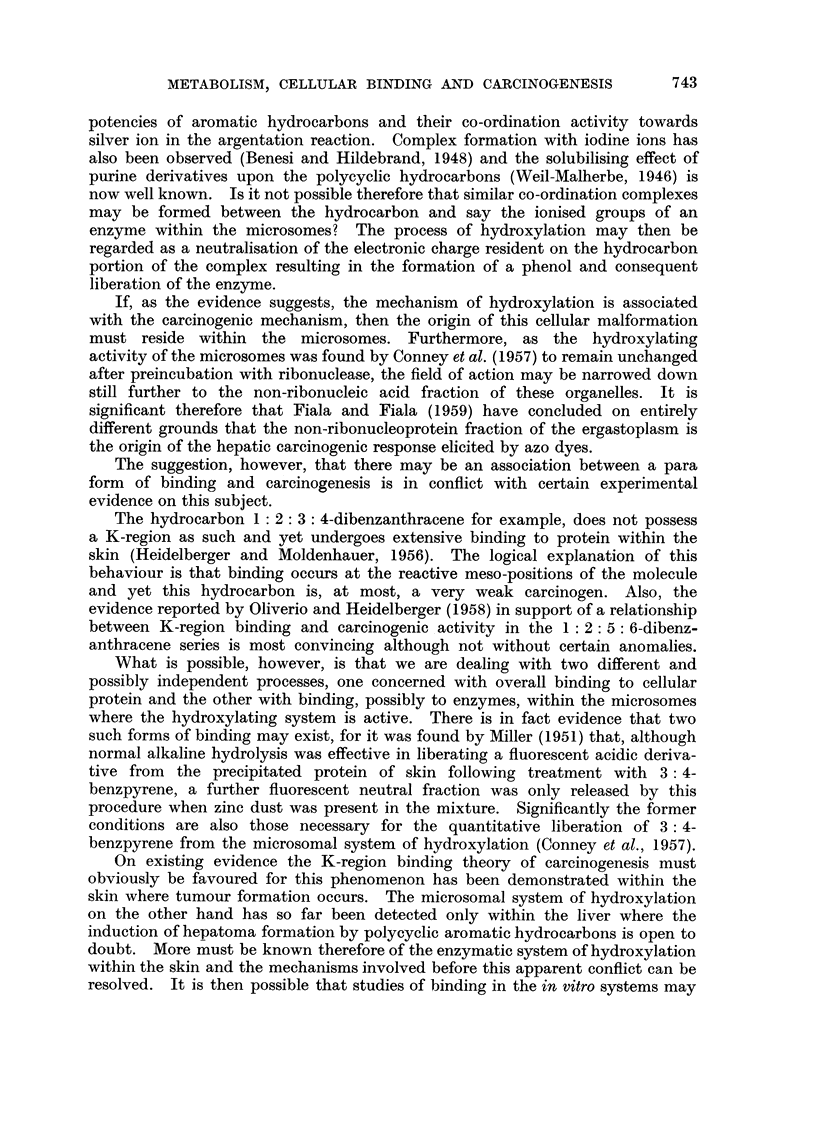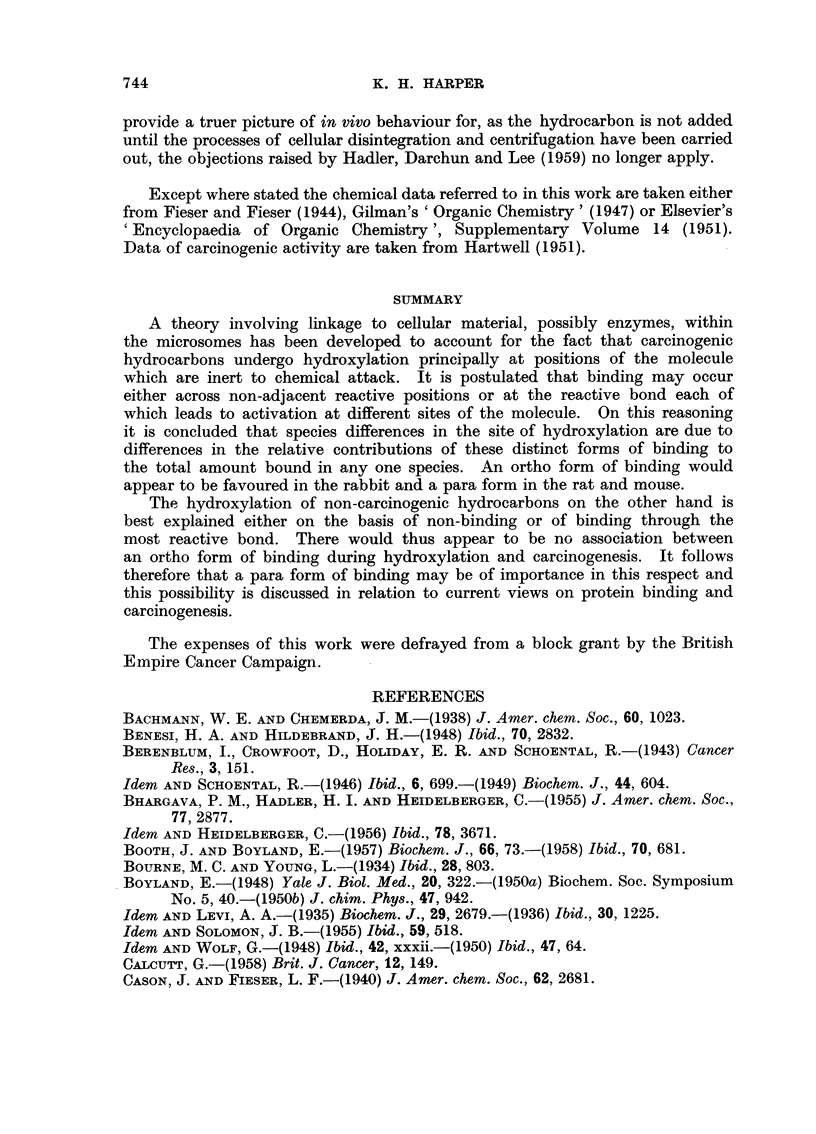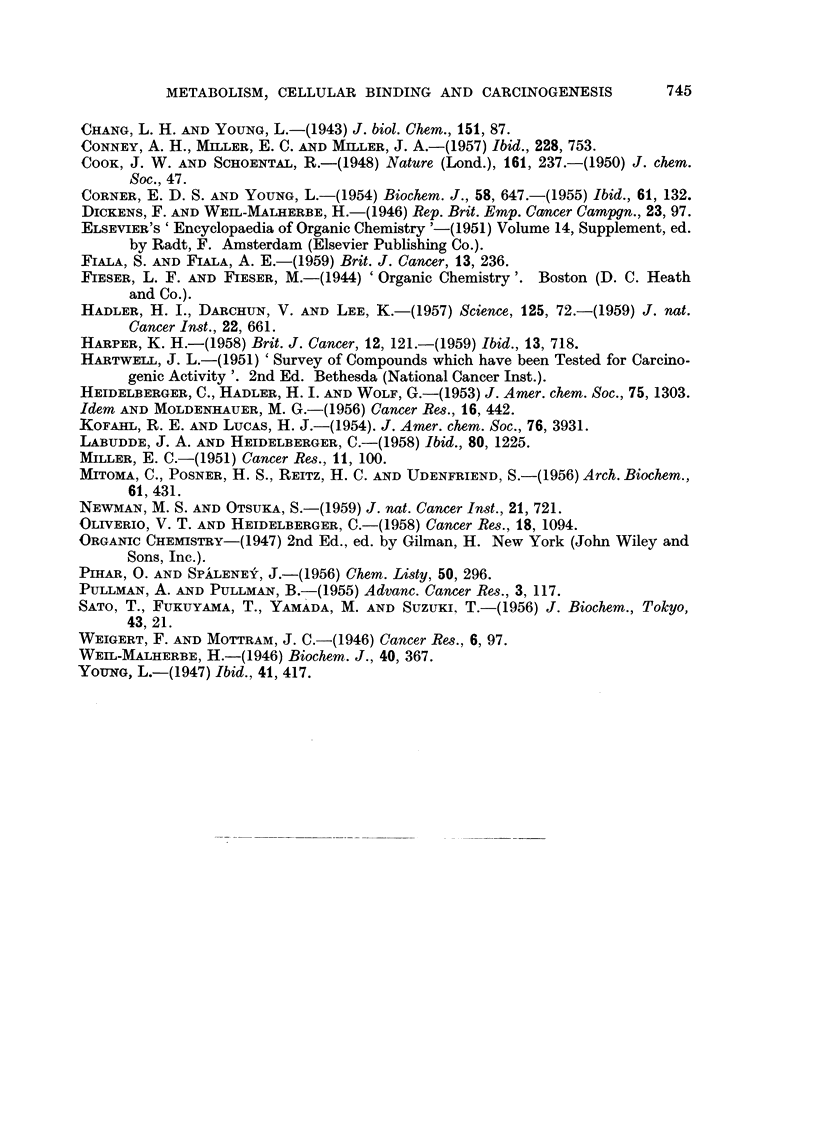# The Polycyclic Hydrocarbons: Metabolism, Cellular Binding and Carcinogenesis

**DOI:** 10.1038/bjc.1959.82

**Published:** 1959-12

**Authors:** K. H. Harper


### THE POLYCYCLIC HYDROCARBONS: METABOLISM, CELLULAR BINDING AND CARCINOGENESIS

K. H. HARPER

*From the Department of Cancer Research, Mount Vernon Hospital and the Radium Institute, Northwood, Middlesex*

Received for publication October 31, 1959

THE data reported in the preceding communication (Harper, 1959) have led to the conclusion that the only important difference between the metabolisms of carcinogenic and non-carcinogenic hydrocarbons lies in the positions of the molecule at which hydroxylation initially occurs. Two possible explanations of this difference were considered to be :

(*a*) That the reactive centres of carcinogenic hydrocarbons are initially blocked by cellular material ; or

(*b*) that different mechanisms of hydroxylation are operative during the metabolism of carcinogenic and non-carcinogenic hydrocarbons respectively.

The mechanism of aromatic hydroxylation is a subject which has received much attention in recent years but so far the investigations have been confined to only two members of the polycyclic hydrocarbon series, namely, naphthalene and 3 : 4-benzpyrene. Fortunately for the purpose of this discussion, however, these may be regarded as typical members of the non-carcinogenic and carcinogenic hydrocarbons respectively.

It was first shown by Mitoma, Posner, Reitz and Udenfriend (1956) that an enzyme system capable of hydroxylating a variety of organic compounds was able to effect the *in vitro* conversion of naphthalene into 1-naphthol (but not 2-naphthol) and a dihydrodiol-like compound which yielded 1-naphthol on acid hydrolysis. The system was located in the microsomal fraction of liver homogenate (but not of brain, kidney, lung and muscle) and required reduced triphosphopyridine nucleotide and oxygen for activity.

A similar enzyme system was found by Conny, Miller and Miller (1957) to effect the hydroxylation of 3 : 4-benzpyrene and the products thus obtained were identical to those yielded during *in vivo* metabolism of the hydrocarbon. The " hydroxylase " was similarly located in the liver microsomes and, although both reduced tri- and diphosphopyridine nucleotides (TPNH and DPNH) and oxygen were required for maximal activity, high activity was supported by TPNH and oxygen alone. The system differed from that of Mitoma *et al.* (1956) however in not being inhibited by the metal binding compounds $\alpha$, $\alpha'$-dipyridyl and *o*-phenanthroline.

Further investigation of the naphthalene hydroxylating system by Booth and Boyland (1957, 1958) confirmed the findings of Mitoma *et al.* with respect to the cellular distribution and requirements of the system but it was found that neither $\alpha$, $\alpha'$-dipyridyl nor *o*-phenanthroline inhibited activity to any great extent. Also, in like manner to the benzpyrene hydroxylating system, inhibition was not observed with either cyanide or cysteine but strong inhibition occurred in the presence of *p*-chloromercuribenzoate suggesting the involvement of sulphydryl groups.

On this evidence therefore it appears probable that the same enzyme, or closely related enzymes with the same co-enzyme requirements, are responsible for the hydroxylation of naphthalene, a non-carcinogen, and 3 : 4-benzpyrene, a potent carcinogen. If such is the case it is unlikely that such closely related systems operate by different mechanisms.

This brings us back to the alternative possibility therefore, of an initial blocking of the reactive centres of the carcinogen by cellular material. The problem in this case is to determine at what region of the molecule such binding is likely to occur, for reference to Table II of the preceding communication (Harper, 1959) reveals the presence of both reactive carbon atoms and reactive bonds although these coincide in certain cases.

The possibility of binding across reactive non-adjacent carbon atoms was considered by Dickens and Weil-Malherbe (1946) who compared metabolic hydroxylation with the chemical method used most successfully in the synthesis of certain metabolic phenols, i.e. sulphonation of the meso-quinone followed by reduction and alkali fusion. An attempted synthesis of 8-benzpyrenol starting with the 5 : 10-quinone was unsuccessful owing to failure at the reduction stage but none the less Dickens and Weil-Malherbe considered that this chemical evidence tallies well with the conception that the most reactive centres of the hydrocarbon molecule are blocked with a cellular constituent and that oxidation then occurs at the most reactive centres remaining.

The other possibility of binding at the reactive bond of the hydrocarbon was proposed by Boyland (1948, 1950*a*) and a similar conclusion concerning the formation of 3-chrysenol from chrysene was arrived at by Berenblum and Schoental (1949). The reactive bond corresponds, in most cases, to the so-called K-region of the molecule and great significance is attached to this proposal when considered in relation to the evidence obtained by the Heidelberger school on the binding of hydrocarbons to protein within the skin. In the case of one hydrocarbon, 1 : 2 : 5 : 6-dibenzanthracene, it has actually been established that about 25 per cent of the bound total is attached through both mono- and di-amido linkages in one K-region (Bhargava and Heidelberger, 1956) so that here is experimental evidence in support of the Boyland hypothesis.

A theoretical treatment of this problem by Pullman and Pullman (1955) was based upon the somewhat arbitrary assumption that the hydrocarbon, bound through its K-region, then exists in an ortho-quinonoidal configuration. In the example selected, 1 : 2-benzanthracene, it was calculated that the reactive centre of the hydrocarbon bound in this way then resides on the 3′ carbon atom and not the metabolic 4′ position. This difficulty was overcome by postulating the formation of an epoxide across the 3′–4′ bond which then undergoes hydrolysis under enzymatic control to yield the 3′–4′ dihydrodiol. Finally the elements of water are split off leaving the hydroxyl group in the 4′ position.

Despite the obvious limitations of such a hypothetical treatment it will be seen that both these proposals, one involving a para and the other an ortho form of binding, are unable in themselves to account for the formation of more than one phenol from the same hydrocarbon. Indeed, the mechanism proposed by Pullman and Pullman (1955) leads to the formation of the same phenol as that obtained from the para-quinone by the described chemical synthesis. Examples of phenolic metabolites are shown in Fig. 1 and it will be seen that

FIG. 1.—Phenolic metabolites of the polycyclic hydrocarbons.

these can be classified on the basis of the structural relationship they bear to one another into two distinct groups designated as A and B in the diagram. What is proposed by the author therefore is that the hydrocarbon may undergo both ortho and para forms of binding during hydroxylation and that in these two states different secondary positions of the molecule become activated. Such a proposal is not entirely speculative for only 25 per cent of the total bound 1 : 2 : 5 : 6-dibenzanthracene could be accounted for by binding in the K-region (Bharagava and Heidelberger, 1956). The remaining 75 per cent is presumably bound at some other region of the molecule. Also 1 : 2 : 3 : 4-dibenzanthracene does not possess an active K-region and yet is bound to an even greater extent than the 1 : 2 : 5 : 6-isomer (Heidelberger and Moldenhauer, 1956). In this case evidence of binding in the reactive meso-positions has in fact been obtained (Oliverio and Heidelberger, 1958).

The essential problem then is to determine which form of binding is responsible for the formation of Group A phenols and which form for Group B. For this purpose the effect of both forms of binding will be considered in relation to the products yielded by individual hydrocarbons. As the exact mode of binding is as yet unknown the hydrocarbon will be assumed to form an addition type of complex as proposed by Boyland (1950*a*). In such compounds the nuclear bond system is that of the ortho- or para-quinone although there is little contribution of the bond linkages to the resonance energy of the system. Should the binding prove to be of a different nature however, it is anticipated that similar considerations will apply.

### 1 : 2 : 5 : 6-*Dibenzanthracene*

Three phenolic metabolites of this hydrocarbon have now been identified, the 4′ : 8′-dihydroxy derivative from the rat and mouse (Cason and Fieser, 1940) and the 2′-hydroxy and 2′ : 6′-dihydroxy derivatives from the rabbit (Labudde and Heidelberger, 1958). In this instance where the molecule is symmetrical about a central axis, it is to be anticipated that binding across this central axis (I) will assure equal activation in the two halves of the molecule

I

II

III

(cf. the sulphonation of 1 : 2 : 5 : 6-dibenz-9 : 10-anthraquinone). Monohydroxylation would not therefore be expected. If on the other hand, binding were to occur at one of the two K-regions (II) then in this instance, where the activating group is asymmetrically situated, it is unlikely that two positions of the molecule would become activated to the same extent. Consequently the formation of a monohydroxylated derivative only would be favoured. In the event of binding at both K-regions, however, the activation in both halves of the molecule would then be equal and dihydroxylation would most probably take place.

It will be seen therefore that the formation of both mono- and di-hydroxylated products in the rabbit is best explained on the basis of an ortho form of binding at one and both K-regions respectively. Conversely the formation of a dihydroxylated—but not a monohydroxylated—derivative in the rat and mouse is consistent with a para form of binding across the reactive meso-positions. The inference is therefore that species differences in metabolism are due to differences in the region of the molecule at which linkage to cellular material occurs during hydroxylation in these species. If this reasoning is correct an ortho form of binding would appear to be favoured in the rabbit and a para form in the rat and mouse. The question immediately arises, however, as to why the mouse does not yield a mixture of phenolic metabolites for K-region binding in this species is now an experimentally established fact. One obvious answer is that the 2′- and 2′ : 6′-hydroxylated compounds are formed but in amounts so small that they have so far escaped detection. This behaviour would then fall into line with that of 3 : 4-benzpyrene for which only a quantitative difference in metabolism has been reported (see later). A possible explanation of this is that the bound complex II behaves as a 2 : 3-disubstituted phenanthrene derivative and as such is particularly susceptible to oxidation at the reactive 9–10 bond, corresponding to the 7–8 bond of dibenzanthracene. Consistent with this hypothesis is the formation within the tissues of the 3 : 4-quinone (Heidelberger, Hadler and Wolf, 1953) and 2-phenylphenanthrene-3 : 2′-dicarboxylic acid (Bhargava, Hadler and Heidelberger, 1955).

1 : 2-*Benzanthracene*

IV V

Application of this reasoning to 1 : 2-benzanthracene leads to the conclusion that, by analogy, the 4′-hydroxy metabolite, a Group A phenol, is formed as a result of binding across the reactive meso-positions (IV). The formation of 2′-hydroxy-1 : 2-benzanthracene is therefore to be anticipated as a consequence of binding in the K-region (V) and it is significant that the evidence reported in the preceding publication is consistent with the formation of an additional phenolic metabolite. It would obviously be of great interest to determine the major site of metabolism in the rabbit where, according to theory, a K-region binding of hydroxylation is favoured. Investigations are already in hand to test this proposal.

### 3 : 4-*Benzpyrene*

The formation of six different phenolic metabolites from 3 : 4-benzpyrene has now been reported, 5-benzpyrenol (Pihar and Spáleneý, 1956), 8-benzpyrenol (Berenblum, Crowfoot, Holiday and Schoental, 1943), 10-benzpyrenol (Berenblum and Schoental, 1946), 5 : 8- and 5 : 10-dihydroxy-benzpyrenes (Conney *et al.*, 1957) and an unidentified phenol designated as the $F_1$ metabolite (Weigert and Mottram, 1946 ; Harper, 1958).

VI

VII

VIII

The ease with which 3 : 4-benzpyrene undergoes oxidation to a mixture of 5 : 8-, 5 : 10-, and possibly 6 : 7-quinones (Cook and Schoental, 1950) suggests that the molecule readily assumes the nuclear bond structures VI–VIII. It is possible then that binding may occur across the 5 : 8-, 5 : 10-, and 6 : 7- (K-region) positions of the molecule, each of which leads to the formation of a different phenol. By analogy with 1 : 2 : 5 : 6-dibenzanthracene an ortho form of binding in the K-region (VI) may be expected to activate the 10-position and it is significant that 10-benzpyrenol, a Group B phenol, is formed to a greater extent in the rabbit than in the rat and mouse (Berenblum and Schoental, 1946).

As the para configuration of structure VII is analogous to that of a true meso-quinone it is then possible that binding across the 5,10 positions would result in activation of the 8-position (Group A). By elimination, formation of the $F_1$ metabolite may then be attributed to binding in the 5,8 positions.

An alternative treatment, however, is to regard the K-region bound structure VI as a 6 : 7-disubstituted chrysene. On this interpretation position 2 of the chrysene molecule, corresponding to the 1-position of benzpyrene, would be expected to be most active and indeed may be expected to represent a major site of hydroxylation. It is possible therefore that the $F_1$ derivative is 1-benzpyrenol and consistent with this hypothesis is the fact that $F_1$ is undoubtedly the major product of hydroxylation during the first few hours following injection of 3 : 4-benzpyrene (Weigert and Mottram, 1946 ; Harper, 1958).

If structure VI does in fact exhibit the normal chemical reactivity of chrysene a further stage of binding may occur at the reactive 1–2 bond (IX) and, by analogy with chrysene (see later), lead to the formation of 10-benzpyrenol.

IX

The other phenolic metabolites of benzpyrene, 5-benzpyrenol and the 5 : 8- and 5 : 10-dihydroxy derivatives, differ from the others so far considered in that hydroxyl groups have entered the molecule in the chemically reactive positions. A possible explanation of this is that hydroxylation may occur to a certain extent when the hydrocarbon is in an unbound state. Perhaps significantly the latter compounds have only been detected in *in vitro* studies with the isolated microsomal system in which the normal *in vivo* pattern of cellular binding may not prevail to the same extent.

*Chrysene*

The hydroxylation of chrysene is more difficult to interpret for the 3-chrysenol rat metabolite (Berenblum and Schoental, 1949) may be regarded as either a Group A or Group B phenol. By analogy with other hydrocarbons an ortho form of binding in the K-region (X) must be favoured in this instance. A para form of binding across the reactive 2,8 positions (XI) on the other hand may be expected to give rise to a symmetrical dihydroxylated derivative.

X

XI

20-*Methylcholanthrene*

XII

XIII

The two possible modes of binding for 20-methylcholanthrene are shown in XII and XIII. By analogy with other hydrocarbons these may be expected to activate the 2 and 4 positions respectively. The ortho bound structure, XII, however, may be regarded as a disubstituted acenaphthene derivative and as such would be more prone to oxidation at the 15–16 bond of the pentacyclic ring (cf. the metabolism of acenaphthene, Chang and Young, 1943). The evidence reported in the preceding paper suggests that this does not occur to any appreciable extent and a para form of binding (XIII) across the 11, 14 positions is therefore favoured. A possible structure for the phenolic metabolite isolated in that work is therefore 4-hydroxy-20-methylcholanthrene.

So far these theoretical considerations have been confined to the carcinogenic members of the polycyclic hydrocarbons. As was pointed out in the preceding publication (Harper, 1959) the products obtained from non-carcinogenic hydrocarbons are consistent with the view that hydroxylation occurs at the most reactive positions of the molecule and perhydroxylation at the most reactive bonds although the perhydroxylation of phenanthrene at the 1–2 bond in the rabbit is an obvious exception to this generalisation. This behaviour may be interpreted as indicating that the non-carcinogenic members are metabolised primarily when in an unbound state and would be in accordance with the conclusion of Heidelberger and Moldenhauer (1956) that non-carcinogenic hydrocarbons do not undergo binding to protein to any appreciable extent. The evidence of Hadler, Darchun and Lee (1957), however, would suggest that the non-carcinogens may go through a transient protein-bound phase of at least the same order of magnitude as that observed with the carcinogens. The findings of Calcutt (1958) are not only consistent with this view but also show that binding may occur in other tissues apart from the skin. It is of interest therefore to speculate upon the effects of ortho and para forms of binding on the reactivities of this class of compounds.

*Naphthalene*

XIV

XV

A variety of hydroxylated products, 1- and 2-naphthol (Bourne and Young, 1934; Corner and Young, 1954, 1955), 1 : 2-dihydroxy-naphthalene (Corner and Young, 1954, 1955), 1 : 2-dihydroxy-1 : 2-dihydro-naphthalene (Young, 1947) and 1-hydroxy-1 : 2-dihydro-naphthalene (Boyland and Solomon, 1955) are formed during the metabolism of this hydrocarbon. In all cases, however, attack is at the 1–2 bond and any form of binding must therefore activate this region of the molecule.

The two possible modes of binding for naphthalene are shown in XIV and XV. It is highly improbable that either of these lead to activation in the unsaturated benzene ring and the only possible means of activation of the metabolic bond is therefore via the ortho-bound structure XIV, as proposed by Boyland (1950*b*).

*Anthracene*

XVI

XVII

Unlike naphthalene the reactive carbon atoms of anthracene do not coincide with the reactive bond. If, as concluded earlier, hydroxylation of the unbound hydrocarbon occurs at the reactive positions of the molecule then the derivative 9 : 10-dihydroxyanthracene (or 9 : 10-anthraquinone) is to be anticipated as a result of this process. There is in fact evidence that the 9 : 10-quinone is excreted as a metabolite of anthracene in the urine (Boyland and Levi, 1936) although the possibility of this being present as an impurity in the administered hydrocarbon was not discounted in that work. Since then however it has been reported that administered 9 : 10-anthraquinone is itself subjected to hydroxylation (Sato, Fukuyama, Yamada and Suzuki, 1956) so that the quinone isolated by Boyland and Levi was probably a true product of hydroxylation and not due to impurity. The major point of attack, however, is at the 1–2 bond resulting in the formation of 1 : 2-dihydroxy-1 : 2-dihydro-anthracene (Boyland and Levi, 1935). What must be decided, therefore, is whether the ortho or para bound structures (XVI and XVII) above are characterised by any marked reactivity at the 1–2 bond.

Unfortunately little is known of the reactivity of the 1,2 addition compounds although the fact that the perhydroxylation of anthracene with Criegie's reagent (Cook and Schoental, 1948) leads to the formation of the 1 : 2 : 3 : 4-tetrahydroxy-1 : 2 : 3 : 4-tetrahydro derivative (XVIII) and not the 1 : 2-dihydrodiol (XIX) suggests that marked activation of the 3–4 bond may occur, assuming the course of the reaction to be as shown.

XIX XVIII

The process of para-blocking in the 9,10 position, however, as with hydrogenation and quinone formation, leads to the formation of particularly stable compounds in which the reactivity of the molecule as a whole is greatly reduced by comparison with that of anthracene. Addition at these points therefore is to be regarded as a deactivating rather than activating mechanism.

An ortho form of binding (XVI), as proposed by Boyland and Wolf (1950), is therefore favoured in this instance although the alternative para form is not entirely discounted.

*Phenanthrene*

Phenanthrene is the sole non-carcinogenic hydrocarbon for which a species difference in the site of hydroxylation has been reported, the 9 : 10-dihydrodiol being formed in the rat and mouse and a mixture of the 9 : 10- and 1 : 2-dihydrodiols in the rabbit (Boyland and Wolf, 1948, 1950). The fact that the carbon atoms comprising the highly reactive 9–10 bond are themselves the reactive positions makes it unlikely that perhydroxylation at this bond is due to binding at some other region of the molecule.

XX

The additional formation of the 1 : 2-dihydrodiol in the rabbit, however, may be attributed to binding across the reactive 9–10 bond (XX), as proposed by Boyland and Wolf (1950), and provides further support for the suggestion that an ortho form of binding is favoured in the rabbit. The fact that 9 : 10-dihydrophenanthrene undergoes Friedel and Crafts' acylation exclusively in the 2-position is evidence that such addition may have a directive influence.

To summarise these proposals, therefore, it can be said that the metabolism of non-carcinogenic hydrocarbons is best explained on the assumption that hydroxylation occurs when they are either in a free state or bound to cellular material through their most reactive bond. The metabolism of carcinogenic members on the other hand would suggest that hydroxylation occurs principally after binding and the formation of different phenols from the same hydrocarbon is then explainable if it is postulated that binding may occur either at the reactive bond or reactive positions of the molecule. On such reasoning it is tentatively concluded that species differences in the site of hydroxylation are due to differences in the relative contributions of these distinct forms of binding to the total amount bound in any one species. An ortho form of binding at the reactive bond would appear to be favoured in the rabbit and a para form across the reactive positions in the rat and mouse.

A critical test of these proposals is the verification that the reactive centres of the ortho and para addition products, or perhaps those of the derived ortho- and para-quinonoidal configurations, do in fact correspond to those attacked during metabolism. Furthermore, as the binding data referred to in this discussion has been obtained from skin, not only must it be established that the same system of microsomal hydroxylation is operative within this tissue but also that the hydrocarbon is actually subjected to binding during hydroxylation in the system. There is evidence in fact that the latter condition is attained, for it was found by Conney *et al.* (1957) that mild alkaline hydrolysis (methanolic potassium hydroxide at 3° C. for 24 to 48 hours) was necessary for the quantitative liberation of 3 : 4-benzpyrene after addition to the *in vitro* hydroxylating system. Further investigation is obviously required to establish this point and other limitations will doubtless be exposed when metabolic studies are extended to other hydrocarbons and other species. The proposals are not put forward in any dogmatic sense, therefore, but as a possible explanation of certain known facts concerning the biochemical hydroxylation of polycyclic hydrocarbons, an explanation which may be checked by direct experimentation.

The final question to be considered is whether these theoretical proposals provide any indication that the process of hydroxylation is associated with the carcinogenic response elicited by certain hydrocarbons. If the non-carcinogenic members of this series do in fact undergo hydroxylation when in an unbound state then an obvious conclusion is that it is the binding, either ortho or para, of the carcinogens which is a contributing factor in carcinogenesis. If, however, the non-carcinogens are first subjected to binding prior to hydroxylation then, as theoretical considerations suggest that this occurs at the reactive bond rather than across the reactive positions, this would appear to exclude the ortho form of binding from the carcinogenic mechanism. Furthermore, the proposal that species differences in metabolism between the rabbit and rat and mouse respectively may be attributed to the greater contribution of ortho binding in the former species is inversely paralleled by the susceptibility of these species towards the induction of carcinogenesis by the hydrocarbons. 1 : 2 : 5 : 6-Dibenzanthracene, for example, is a potent carcinogen for the rat and mouse but is without activity in the rabbit. If the theory is correct, therefore, there would appear to be no association between the ortho or K-region binding of hydroxylation and carcinogenesis. It is tempting to speculate then that the para form of binding may be an important factor governing the carcinogenic response and certain evidence may be cited in support of this view. Thus the blocking of the para-positions in compounds such as cis-9 : 10-dimethyl-9 : 10-dihydro-1 : 2 : 5 : 6-dibenzanthracene and 9 : 10-dimethyl-1 : 2-benzanthracene-$\alpha$, $\beta$-endo-succine acid is accompanied by a marked reduction in the carcinogenic potencies of the parent hydrocarbons from which these are derived. Such behaviour may be interpreted as an indication that substitution in the meso-positions of these hydrocarbons prevents the process of para-binding during hydroxylation. Also, 9 : 10-dimethyl-1 : 2-benzanthracene is considerably more carcinogenic than is the parent 1 : 2-benzanthracene and this behaviour is reflected in the reactivities of these compounds towards the para-addition of maleic anhydride, this occurring more readily with the dimethyl derivative (Bachmann and Chemerda, 1938; Newman and Otsuka, 1959). In other words the effect of methyl substitution in the 9 : 10-positions of 1 : 2-benzanthracene, and indeed in those of anthracene, is to facilitate the process of para-addition at these points. In this case, however, we are dealing with a specific type of cyclic dienophile addition and it is not surprising therefore that the analogy breaks down when applied to the whole range of polycyclic hydrocarbons. What is envisaged biologically rather is an addition type of complex arising from combination of the carcinogen either with two molecules or with distal groups of the same molecule so that it constitutes in effect a cross linkage between the two. As was stated earlier the exact mode of linkage is as yet unknown. Boyland (1950*a*) has suggested a simple covalent type of addition whilst Pullman and Pullman (1955) prefer the quinonoidal type of linkage. A third type of addition not yet considered, however, is that of co-ordination. The latter phenomenon was investigated by Kofahl and Lucas (1954) who reported a fair degree of correlation between the carcinogenic potencies of aromatic hydrocarbons and their co-ordination activity towards silver ion in the argentation reaction. Complex formation with iodine ions has also been observed (Benesi and Hildebrand, 1948) and the solubilising effect of purine derivatives upon the polycyclic hydrocarbons (Weil-Malherbe, 1946) is now well known. Is it not possible therefore that similar co-ordination complexes may be formed between the hydrocarbon and say the ionised groups of an enzyme within the microsomes? The process of hydroxylation may then be regarded as a neutralisation of the electronic charge resident on the hydrocarbon portion of the complex resulting in the formation of a phenol and consequent liberation of the enzyme.

If, as the evidence suggests, the mechanism of hydroxylation is associated with the carcinogenic mechanism, then the origin of this cellular malformation must reside within the microsomes. Furthermore, as the hydroxylating activity of the microsomes was found by Conney *et al.* (1957) to remain unchanged after preincubation with ribonuclease, the field of action may be narrowed down still further to the non-ribonucleic acid fraction of these organelles. It is significant therefore that Fiala and Fiala (1959) have concluded on entirely different grounds that the non-ribonucleoprotein fraction of the ergastoplasm is the origin of the hepatic carcinogenic response elicited by azo dyes.

The suggestion, however, that there may be an association between a para form of binding and carcinogenesis is in conflict with certain experimental evidence on this subject.

The hydrocarbon 1 : 2 : 3 : 4-dibenzanthracene for example, does not possess a K-region as such and yet undergoes extensive binding to protein within the skin (Heidelberger and Moldenhauer, 1956). The logical explanation of this behaviour is that binding occurs at the reactive meso-positions of the molecule and yet this hydrocarbon is, at most, a very weak carcinogen. Also, the evidence reported by Oliverio and Heidelberger (1958) in support of a relationship between K-region binding and carcinogenic activity in the 1 : 2 : 5 : 6-dibenzanthracene series is most convincing although not without certain anomalies.

What is possible, however, is that we are dealing with two different and possibly independent processes, one concerned with overall binding to cellular protein and the other with binding, possibly to enzymes, within the microsomes where the hydroxylating system is active. There is in fact evidence that two such forms of binding may exist, for it was found by Miller (1951) that, although normal alkaline hydrolysis was effective in liberating a fluorescent acidic derivative from the precipitated protein of skin following treatment with 3 : 4-benzpyrene, a further fluorescent neutral fraction was only released by this procedure when zinc dust was present in the mixture. Significantly the former conditions are also those necessary for the quantitative liberation of 3 : 4-benzpyrene from the microsomal system of hydroxylation (Conney *et al.*, 1957).

On existing evidence the K-region binding theory of carcinogenesis must obviously be favoured for this phenomenon has been demonstrated within the skin where tumour formation occurs. The microsomal system of hydroxylation on the other hand has so far been detected only within the liver where the induction of hepatoma formation by polycyclic aromatic hydrocarbons is open to doubt. More must be known therefore of the enzymatic system of hydroxylation within the skin and the mechanisms involved before this apparent conflict can be resolved. It is then possible that studies of binding in the *in vitro* systems may provide a truer picture of *in vivo* behaviour for, as the hydrocarbon is not added until the processes of cellular disintegration and centrifugation have been carried out, the objections raised by Hadler, Darchun and Lee (1959) no longer apply.

Except where stated the chemical data referred to in this work are taken either from Fieser and Fieser (1944), Gilman's ' Organic Chemistry ' (1947) or Elsevier's ' Encyclopaedia of Organic Chemistry ', Supplementary Volume 14 (1951). Data of carcinogenic activity are taken from Hartwell (1951).

### SUMMARY

A theory involving linkage to cellular material, possibly enzymes, within the microsomes has been developed to account for the fact that carcinogenic hydrocarbons undergo hydroxylation principally at positions of the molecule which are inert to chemical attack. It is postulated that binding may occur either across non-adjacent reactive positions or at the reactive bond each of which leads to activation at different sites of the molecule. On this reasoning it is concluded that species differences in the site of hydroxylation are due to differences in the relative contributions of these distinct forms of binding to the total amount bound in any one species. An ortho form of binding would appear to be favoured in the rabbit and a para form in the rat and mouse.

The hydroxylation of non-carcinogenic hydrocarbons on the other hand is best explained either on the basis of non-binding or of binding through the most reactive bond. There would thus appear to be no association between an ortho form of binding during hydroxylation and carcinogenesis. It follows therefore that a para form of binding may be of importance in this respect and this possibility is discussed in relation to current views on protein binding and carcinogenesis.

The expenses of this work were defrayed from a block grant by the British Empire Cancer Campaign.

### REFERENCES

BACHMANN, W. E. AND CHEMERDA, J. M.—(1938) *J. Amer. chem. Soc.*, **60**, 1023.
BENESI, H. A. AND HILDEBRAND, J. H.—(1948) *Ibid.*, **70**, 2832.
BERENBLUM, I., CROWFOOT, D., HOLIDAY, E. R. AND SCHOENTAL, R.—(1943) *Cancer Res.*, **3**, 151.
*Idem* AND SCHOENTAL, R.—(1946) *Ibid.*, **6**, 699.—(1949) *Biochem. J.*, **44**, 604.
BHARGAVA, P. M., HADLER, H. I. AND HEIDELBERGER, C.—(1955) *J. Amer. chem. Soc.*, **77**, 2877.
*Idem* AND HEIDELBERGER, C.—(1956) *Ibid.*, **78**, 3671.
BOOTH, J. AND BOYLAND, E.—(1957) *Biochem. J.*, **66**, 73.—(1958) *Ibid.*, **70**, 681.
BOURNE, M. C. AND YOUNG, L.—(1934) *Ibid.*, **28**, 803.
BOYLAND, E.—(1948) *Yale J. Biol. Med.*, **20**, 322.—(1950*a*) Biochem. Soc. Symposium No. 5, 40.—(1950*b*) *J. chim. Phys.*, **47**, 942.
*Idem* AND LEVI, A. A.—(1935) *Biochem. J.*, **29**, 2679.—(1936) *Ibid.*, **30**, 1225.
*Idem* AND SOLOMON, J. B.—(1955) *Ibid.*, **59**, 518.
*Idem* AND WOLF, G.—(1948) *Ibid.*, **42**, xxxii.—(1950) *Ibid.*, **47**, 64.
CALCUTT, G.—(1958) *Brit. J. Cancer*, **12**, 149.
CASON, J. AND FIESER, L. F.—(1940) *J. Amer. chem. Soc.*, **62**, 2681.

Chang, L. H. and Young, L.—(1943) *J. biol. Chem.*, **151**, 87.
Conney, A. H., Miller, E. C. and Miller, J. A.—(1957) *Ibid.*, **228**, 753.
Cook, J. W. and Schoental, R.—(1948) *Nature* (Lond.), **161**, 237.—(1950) *J. chem. Soc.*, 47.
Corner, E. D. S. and Young, L.—(1954) *Biochem. J.*, **58**, 647.—(1955) *Ibid.*, **61**, 132.
Dickens, F. and Weil-Malherbe, H.—(1946) *Rep. Brit. Emp. Cancer Campgn.*, **23**, 97.
Elsevier's ' Encyclopaedia of Organic Chemistry '—(1951) Volume 14, Supplement, ed. by Radt, F. Amsterdam (Elsevier Publishing Co.).
Fiala, S. and Fiala, A. E.—(1959) *Brit. J. Cancer*, **13**, 236.
Fieser, L. F. and Fieser, M.—(1944) ' Organic Chemistry '. Boston (D. C. Heath and Co.).
Hadler, H. I., Darchun, V. and Lee, K.—(1957) *Science*, **125**, 72.—(1959) *J. nat. Cancer Inst.*, **22**, 661.
Harper, K. H.—(1958) *Brit. J. Cancer*, **12**, 121.—(1959) *Ibid.*, **13**, 718.
Hartwell, J. L.—(1951) ' Survey of Compounds which have been Tested for Carcinogenic Activity '. 2nd Ed. Bethesda (National Cancer Inst.).
Heidelberger, C., Hadler, H. I. and Wolf, G.—(1953) *J. Amer. chem. Soc.*, **75**, 1303.
*Idem* and Moldenhauer, M. G.—(1956) *Cancer Res.*, **16**, 442.
Kofahl, R. E. and Lucas, H. J.—(1954). *J. Amer. chem. Soc.*, **76**, 3931.
Labudde, J. A. and Heidelberger, C.—(1958) *Ibid.*, **80**, 1225.
Miller, E. C.—(1951) *Cancer Res.*, **11**, 100.
Mitoma, C., Posner, H. S., Reitz, H. C. and Udenfriend, S.—(1956) *Arch. Biochem.*, **61**, 431.
Newman, M. S. and Otsuka, S.—(1959) *J. nat. Cancer Inst.*, **21**, 721.
Oliverio, V. T. and Heidelberger, C.—(1958) *Cancer Res.*, **18**, 1094.
Organic Chemistry—(1947) 2nd Ed., ed. by Gilman, H. New York (John Wiley and Sons, Inc.).
Pihar, O. and Spáleneý, J.—(1956) *Chem. Listy*, **50**, 296.
Pullman, A. and Pullman, B.—(1955) *Advanc. Cancer Res.*, **3**, 117.
Sato, T., Fukuyama, T., Yamada, M. and Suzuki, T.—(1956) *J. Biochem., Tokyo*, **43**, 21.
Weigert, F. and Mottram, J. C.—(1946) *Cancer Res.*, **6**, 97.
Weil-Malherbe, H.—(1946) *Biochem. J.*, **40**, 367.
Young, L.—(1947) *Ibid.*, **41**, 417.